# Streamlining Skin Regeneration: A Ready-To-Use Silk Bilayer Wound Dressing

**DOI:** 10.3390/gels10070439

**Published:** 2024-06-30

**Authors:** Anabela Veiga, Inês V. Silva, Juliana R. Dias, Nuno M. Alves, Ana L. Oliveira, Viviana P. Ribeiro

**Affiliations:** 1 Universidade Católica Portuguesa, CBQF—Centro de Biotecnologia e Química Fina—Laboratório Associado, Escola Superior de Biotecnologia, Rua Diogo Botelho 1327, 4169-005 Porto, Portugal; s-anveiga@ucp.pt (A.V.); s-ivmsilva@ucp.pt (I.V.S.); vpribeiro@ucp.pt (V.P.R.); 2 LEPABE—Laboratory for Process Engineering, Environment, Biotechnology & Energy, Department of Chemical Engineering, Faculty of Engineering, University of Porto, 4200-465 Porto, Portugal; 3 ALiCE—Associate Laboratory in Chemical Engineering, Faculty of Engineering, University of Porto, Rua Dr. Roberto Frias, 4200-465 Porto, Portugal; 4 Centre for Rapid and Sustainable Product Development, Instituto Politécnico de Leiria, 2430-028 Marinha Grande, Portugal; juliana.dias@ipleiria.pt (J.R.D.); nuno.alves@ipleiria.pt (N.M.A.)

**Keywords:** bilayer, silk sericin, silk fibroin, wound dressing

## Abstract

Silk proteins have been highlighted in the past decade for tissue engineering (TE) and skin regeneration due to their biocompatibility, biodegradability, and exceptional mechanical properties. While silk fibroin (SF) has high structural and mechanical stability with high potential as an external protective layer, traditionally discarded sericin (SS) has shown great potential as a natural-based hydrogel, promoting cell–cell interactions, making it an ideal material for direct wound contact. In this context, the present study proposes a new wound dressing approach by developing an SS/SF bilayer construct for full-thickness exudative wounds. The processing methodology implemented included an innovation element and the cryopreservation of the SS intrinsic secondary structure, followed by rehydration to produce a hydrogel layer, which was integrated with a salt-leached SF scaffold to produce a bilayer structure. In addition, a sterilization protocol was developed using supercritical technology (sCO_2_) to allow an industrial scale-up. The resulting bilayer material presented high porosity (>85%) and interconnectivity while promoting cell adhesion, proliferation, and infiltration of human dermal fibroblasts (HDFs). SS and SF exhibit distinct secondary structures, pore sizes, and swelling properties, opening new possibilities for dual-phased systems that accommodate the different needs of a wound during the healing process. The innovative SS hydrogel layer highlights the transformative potential of the proposed bilayer system for biomedical therapeutics and TE, offering insights into novel wound dressing fabrication.

## 1. Introduction

Chronic wounds are currently a global epidemic, and it is reported that 1–2% of people in developed countries will experience chronic wounds at least once in their lifetime. In the US alone, they impact ~8.5 M people and cost ~28 B USD/year, not accounting for the pain and suffering patients endure, psychologically, physically, and financially [1]. The current clinical gold-standard treatment is skin grafting, which is often limited by wound area, donor source, and costly medical expenses [2]. Wound dressings serve as an alternative to skin grafting by providing a protective barrier and reducing infection risk, particularly in cases where skin grafting may not be feasible or necessary. Traditional wound dressings, such as gauze, non-adherent silicone, silver, hydrocolloids, and foam dressings, are versatile and readily available [3,4,5,6,7]. However, inadequate moisture control and adherence issues highlight the need for advanced solutions, placing the tissue-engineered skin substitutes that unblock the inflammatory stage and promote the gradual refurbishment of injured tissue to advance functional integrity and regeneration as promising alternatives. In this context, different tissue engineering (TE) approaches have been proposed for wound dressing [8,9]. Among them, commercially available natural-based hydrogel wound dressings include alginate (1), chitosan (2), and collagen (3). However, several constraints are also reported: (1) limited hydration and requirement of a secondary dressing [10,11]; (2) limited availability, high risk of batch-to-batch variations and allergic reactions [12,13,14]; and (3) enzymatic degradation leading to rapid loss of dressing stability and shape, risk of pathogen transmission, and high cost [15,16,17]. In this context, bilayer scaffolding strategies [8,9,18,19] are being reported as enhanced wound dressings as a result of their combined properties (one layer can focus on moisture retention and exudate management, while the other could provide mechanical protection or deliver therapeutic agents) [20]. Bilayer wound dressings are able to adapt to the rate of healing or allow on-demand removability to facilitate the healing process [21].

Silk, a naturally occurring protein, has gained approval from the US Food and Drug Administration (FDA) for specific medical applications [22]. Fibroin (SF), the primary constituent of silk, forms the resilient inner core, providing mechanical durability, while sericin (SS) acts as a protective coating on the outer surface [23]. Unlike SF, SS has not been extensively studied as a biomaterial for wound healing. This protein is conventionally discarded with the silk degumming wastewaters (with a content that may be as high as 30 g/L), resulting in a high chemical oxygen demand and environmental pollution [24]. However, recent scientific evidence has showcased the advantageous characteristics of SS, including its biocompatibility, biodegradability, and ability to stimulate collagen production [25], as well as its anti-oxidative [26], anti-tyrosinase [27], anti-inflammatory [28], anticoagulatory [29], antitumor, anti-aging [30], and anti-wrinkle properties [31]. Moreover, SS degrades more rapidly than SF in aqueous solution [32]. Thus, SS has the potential to be used as a temporary barrier against external infections and serves as an induction template to guide the reorganization of skin cells and the subsequent infiltration and integration of host tissues [33,34].

A critical issue in the application of wound dressings or any other type of medical device is the chosen sterilization method, which although essential to reduce wound exposure to microorganisms can negatively affect the integrity of natural biomaterials. Traditional methods of sterilization, such as ethylene oxide (EtO), gamma radiation, electron beam, steam, hydrogen peroxide plasma, and UV radiation, can be excessively harsh to materials, particularly those that are protein-based, resulting in alterations to their structure and properties [ref]. In this regard, supercritical CO_2_ (sCO_2_) has emerged as a promising sterilization agent capable of inactivating both Gram-positive and Gram-negative bacteria in their vegetative forms [35]. The incorporation of small quantities of additives like H_2_O_2_ can further enhance the sterilization effectiveness. The advantages of sCO_2_ sterilization include gentle operating conditions, minimal additive requirements, and excellent permeability of sCO_2_, enabling the preservation of the material’s original physicochemical properties [36,37].

In the present work, a natural and off-the-shelf wound dressing, sterilized using sCO_2_ is presented. The porous bilayer structure is composed of an SS and SF layer and a strongly interconnected SS/SF interface: SS acquires hydrogel-like properties when rehydrated as a result of the processing methodology followed, and it acts as a bioactive layer, providing an active environment for cell growth, as shown in the in vitro tests conducted using HDFs. This layer can absorb wound exudate effectively, as demonstrated by the swelling tests performed, and can sustain continuous release of therapeutic agents directly to the wound site, as evidenced using the albumin fluorescein isothiocyanate conjugate (FITC) model drug. The SF crystalline layer provides the material with structural integrity that results in increased cell growth and protects the SS–wound interface. To our knowledge, this work is the first study to combine SS with preserved properties after extraction for use as a material for wound interaction and SF as the external layer with a structural nature and to explore their structural and bioactive properties to develop an off-the-shelf acellular natural-based wound dressing to be used to promote tissue regeneration.

## 2. Results and Discussion

### 2.1. Structure and Composition

**Figure 1:**
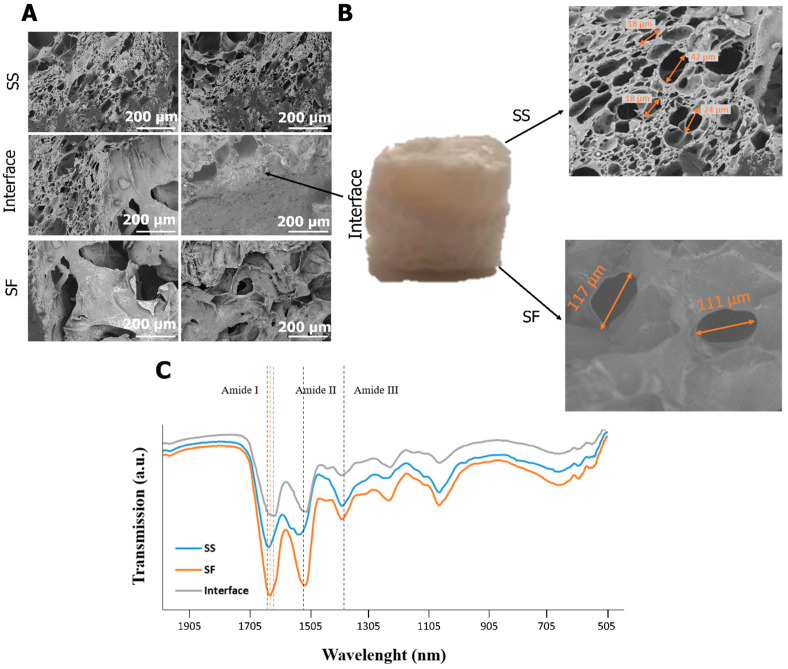
Morphology and typical pore size of the bilayer structures after preparation by SEM (**A**), macroscopic bilayer and SS and SF pore size (**B**), and FTIR spectra (**C**).

Bilayer wound dressings are increasingly being studied, combining multifunctional properties ideal for wound management (antimicrobial, adhesion, homeostasis, and anti-inflammatory properties) with the mechanical stability and tissue integration performance inherent in bilayer structures [8]. In the present work, a silk-based scaffold was developed, taking advantage of the intrinsic properties of SS and SF to obtain a bilayer structure. According to the SEM images (Figure 1A), both structures have high porosity with visible distinctions (Figure 1B) and composition (Figure 1C).

The SF layer is characterized by the presence of larger pores (≈100 µm), as the result of the salt-leaching process [38], while SS presents smaller pores (≈20–40 µm), corresponding to the space occupied by water before the freeze-drying process. At the interface of the material, it is possible to observe that the scaffold layers are well integrated as a result of the penetration of the SS solution into the matrix, creating a compositional gradient (Figure 1A,B). A well-interconnected interface ensures seamless integration between different layers, which contributes to the structural integrity of the material [39].

According to the FTIR spectra (Figure 1C), Amide I (primarily due to the C=O stretching vibration) (1644, 1638, 1624 cm^−1^, for SS, SF, and SS/SF interface, respectively), Amide II (N-H bending and C-N stretching vibrations) (1540, 1520, 1522 cm^−1^, for SS, SF, and SS/SF interface, respectively), and Amide III (from C-N stretching and N-H bending vibrations) (1396 cm^−1^, consistent across all samples) peaks were identified [40,41]. The Amide I peak in SS suggests a mixed secondary structure with less β-sheet content compared to SF (which typically absorbs in the 1610–1640 cm^−1^ range) [41,42]. The β-sheet content of SF is linked to the salt-leaching process due to the integration of sodium chloride into the silk solution [39]. The slight Amide II shift to the right of SF when compared to SS also suggests a difference in the hydrogen bonding pattern and the arrangement of the protein backbone. For the interface, Amide I is closer to the SF peak, possibly due to the influence of this protein structure, while Amide II is very similar to both SS and SF, suggesting a blend or transition of structures at the interface.

**Figure 2:**
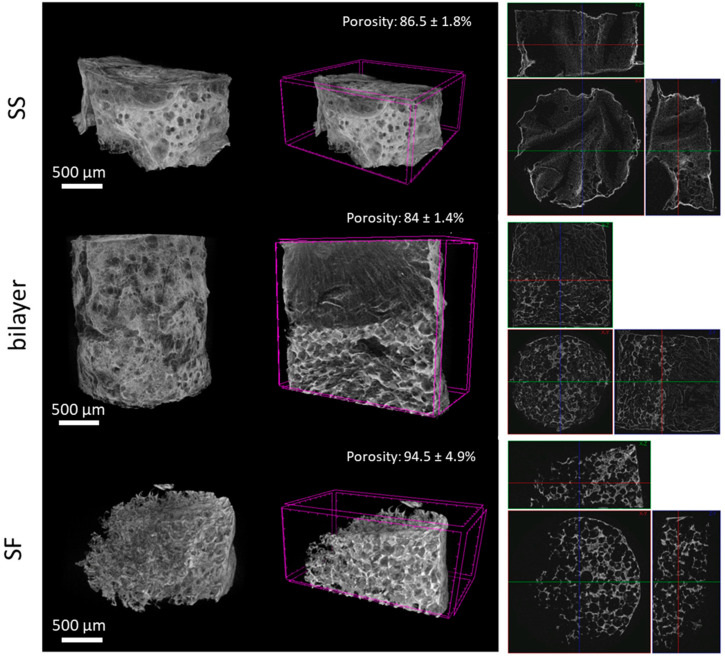
Micro-CT of the bilayer, and individual SS and SF structures.

The porosity and different structure of the scaffolds and bilayer were also evidenced in micro-CT (Figure 2).

According to the 3D assessment of the silk structures (Figure 2), the porosities of the bilayer and the individual SS and SF layers are 84.0 ± 1.4%, 86.5 ± 1.8%, and 94.5 ± 4.9%, respectively. Thus, although the pore sizes are different, as previously discussed (Figure 1A), the porosities are similar.

The uniform pore distribution, homogeneous size, and interconnectivity are in line with the SEM imaging. It is also noteworthy that SF has a brighter color, characteristic of a more crystalline and regular structure, which is in line with the FTIR results, which demonstrate the higher content of the β-sheet structure.

According to the literature, chronic wounds often benefit from dressings with higher porosity to facilitate exudate removal while having moisture retention capability [43,44]. Scaffolds with a porosity range of 60–90% promote cellular activities, enable efficient exchange of oxygen and nutrients, and facilitate the generation of a fresh extracellular matrix (ECM) [45]. Porous structures also enable the development of water vapor-permeable wound dressings that promote proliferation and the regular function of epidermal cells and fibroblasts [46].

In addition, the ideal pore size for wound healing scaffolds typically ranges from 20 to 125 µm [45,47]. Smaller pores (around 20–50 µm) can be beneficial for the initial attachment and proliferation of cells [8,48]. On the other hand, larger pores (100–200 µm) are more suitable for cell migration and vascularization, which are crucial for the formation of new tissue [49,50]. Thus, while the SS small pore size is crucial for the early stages of wound healing, SF’s larger pores in the upper layer in combination with its stable structure (high crystallinity) can provide physical protection to the underlying wound and further promote cellular behavior.

### 2.2. Rehydration Capacity and Porosity

**Figure 3:**
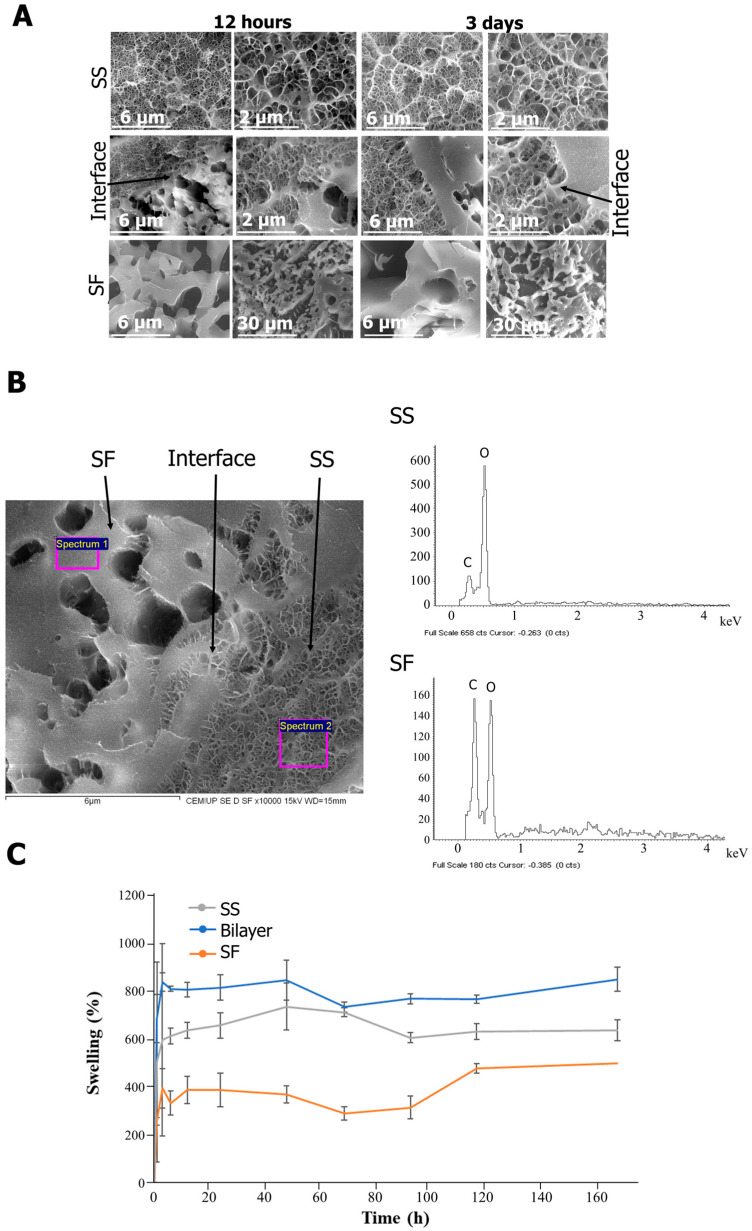
Morphology and porosity of the bilayer structures after rehydration for 12 h and 3 days (cryo−SEM) (**A**) SS, SF, SS/SF interface and SDS elemental analysis of SF and SS layer after rehydration for 12 h, and (**B**) swelling properties of the bilayer structure and individual SS and SF (**C**).

Understanding how SS transforms into a hydrogel-like matrix upon hydration provides insights into its potential as a wound dressing material capable of effectively managing wound exudate. SS’s ability to recover its hydrogel-like properties after hydration was evaluated by cryo-SEM and swelling tests (Figure 3).

Hydration can induce conformational changes in SS which result in a reversion to its gel state [51,52]. After 12 h and 3 days of rehydration, it was shown that the porous structure of SS and SF was maintained, with a well-distributed and homogeneous porosity.

The interface remained stable and well defined after the lyophilization/hydration process. At the SS/SF interface, distinct dual porosity was evident, highlighting the interaction between SS and SF layers. Subsequent analysis revealed a significant decrease in SS penetration into the SF layer with distance from the interface, detectable up to approximately 6 µm (Figure 3A).

EDS allowed the elemental identification of the silk proteins and their purity, by identifying the peaks attributed to carbon (C) and oxygen (O) (Figure 3B) [53,54]. The assessment of the swelling capacity as a result of the rehydration of the bilayer structure showed that maximum swelling is achieved after 3 h of immersion in PBS (Figure 3C). After that period, the scaffold does not suffer significant changes. The swelling capacity of the individual SS layer is superior to the SF layer (reaching 594% and 393% of swelling after 3 h for SS and SF, respectively), resulting in an increased swelling capacity of the bilayer structure (836%). This was expected as a result of SS’s high hydrophilicity when compared to SF [51,55] (Mw: 440–40 KDa when extracted using boiling water), as well as the hydrophilic amino acid residues, such as serine and aspartic acid, present in both proteins, even though SF has an amphiphilic molecular structure. Regarding the bilayer, the lyophilization of the SS hydrogel/salt-leached SF scaffold further increases its porosity and, subsequently, its swelling and exudate absorbance capacity [56].

Wound dressing materials with maximum swelling rates from 50 to 5000% have been reported [57,58] for wounds with different levels of exudate. For exudative wounds, the different pore sizes in bilayer systems can be used to manage wound exudate through distinct swelling behaviors [18]. The smaller pores of SS can manage initial wound exudate, retaining necessary moisture for healing. Upon application at the wound site, some exudate is expected to be absorbed and eventually transferred through the porous layer to avoid wound maceration [59]. As for SF, its crystalline structure also reduces its swelling capacity when compared to the more amorphous SS. According to the literature, pores in the range of 75–150 µm facilitate the transportation and evaporation of excess exudate, preventing over-saturation and aiding in the overall wound healing process [46,60].

### 2.3. Mechanical Properties

**Figure 4:**
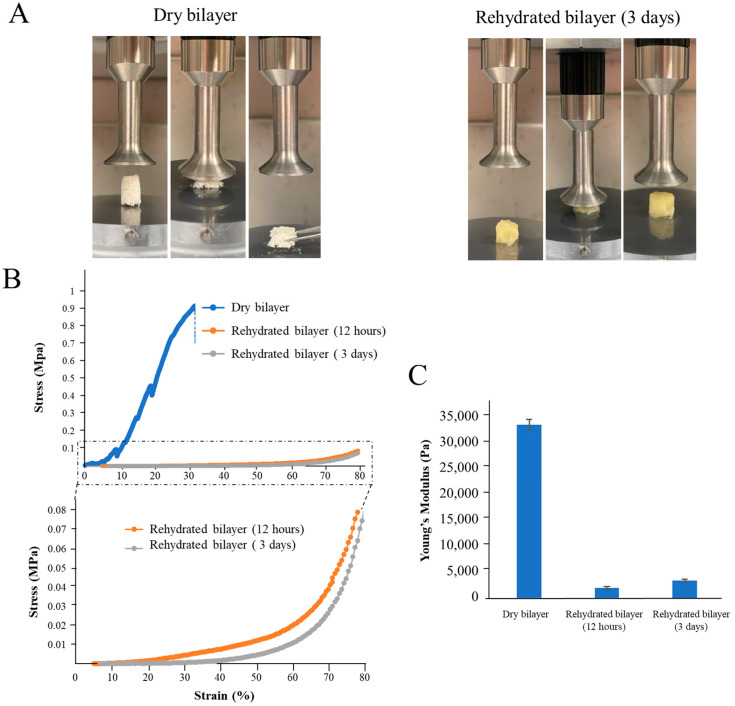
Mechanical properties of the sterile bilayer structures in the dry state and after rehydration for 12 h and 3 days. (**A**) Representative images of the bilayer compression tests performed using the rheometer. (**B**) Stress–strain curves with the enlarged area of the top graph show in detail the curves of the rehydrated bilayer scaffolds. (**C**) Young’s modulus of the bilayer scaffolds.

The mechanical behavior of the bilayers was evaluated before and after rehydration for 3 days (Figure 4).

Although SF has been used to develop different scaffolds, SS is reported to lack the required mechanical properties. Enzymatically crosslinked SS with HRP/H_2_O_2_ leads to the formation of stable hydrogels [33,55], and when cryo-lyophilized, a brittle SS scaffold is formed.

In the dry state, the bilayer scaffold suffered irreversible deformation when submitted to a compression force; however, after rehydration, the scaffold acquired compression-resistance characteristics (Figure 4A), which are typical of spongy-like structures. According to the stress–strain curve (Figure 4B), the dry scaffold fracture occurs at 32% strain. Rupture can occur without any prior noticeable change in the rate of elongation; sometimes, fracturing occurs before yielding [61]. On the other hand, hydrated bilayers exhibit elastic properties (capable of undergoing reversible deformation without permanent damage or yielding) [62]. This can have important implications in the materials as a wound dressing, such as reduced shear forces: elastic dressings can distribute mechanical forces more evenly across the wound site, reducing the occurrence of the shear forces that can be detrimental to wound healing [63]. Moreover, elastic dressings can reduce trauma during removal [64], provide ease of movement to patients, and allow a better adaptation to different wounds [56,65].

According to the results obtained, the materials have Young’s moduli of 33,300 Pa, 2000, and 3425 Pa for the dry state and after immersion for 12 h and 3 days, respectively (no significant differences were registered between 12 h and 3 days of scaffold immersion in PBS) (Figure 4C). This behavior is reported in several scaffolds after hydration as a result of swelling, which reduces the overall stiffness of the 3D construct [66,67,68].

### 2.4. Drug Delivery Capacity

**Figure 5:**
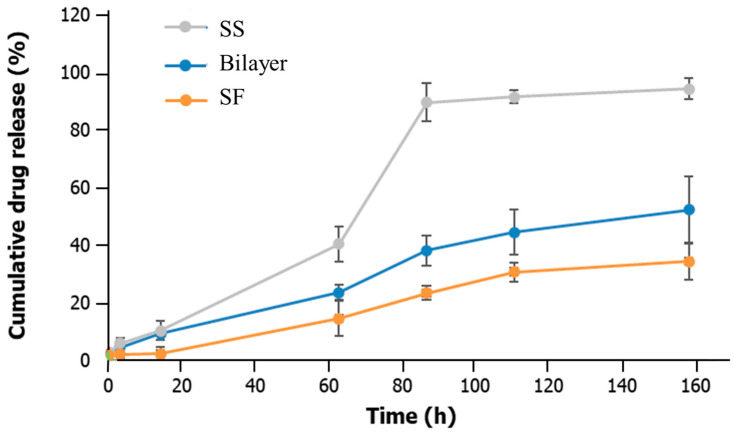
Drug release profile of the bilayer structure and individual SS and SF scaffolds.

The drug delivery capacity of the bilayer and the SS and SF layers was assessed using an albumin-FITC model drug (Figure 5). This protein has binding sites for various substances and is a representation of many therapeutic proteins.

The incorporation efficiency on the scaffolds was 97.60% ± 0.36%, in line with a previously published work that focused on silk protein scaffolds [69]. It was shown that after approximately 92 h, 95%, 52%, and 34% of the albumin-FITC model drug was released from the SS, bilayer, and SF scaffolds. The lowest cumulative drug release of SF can be attributed to its β-sheet structure and larger pore size, which result in a slower or more controlled diffusion and drug release, which is beneficial for sustained treatment [70,71]. This is in line with other works in which salt-leached SF scaffolds enabled fibroblast growth factor (bFGF) release in a sustained fashion (for 2 weeks) [69,72], while a β-sheet SF lyophilized scaffold incorporated with albumin exhibited a slow and controlled release profile of albumin (between 22% and 47% at the 72 h time point) [69]. In contrast, SS, with its smaller pores and higher swelling capacity, released the drug more rapidly. This is suitable for the early stages of wound healing, where an initial drug delivery might be necessary to target inflammation or avoid infection [73].

The drug release behavior of an SS hydrogel combined with H_2_O_2_ was assessed in a published work using an adriamycin hydrochloride (DOX-HCl) model drug. In the initial stage, the burst release of DOX-HCl appeared, and the cumulative drug release was almost proportional to the incubation time [74]. Other studies have also investigated the potential of SS composite constructs for drug release applications. An SS/poly(ethylene glycol) diacrylate (PEGDA) hydrogel was used to load berberine [75], showing an initial burst release during the first hour due to the rapid release of surface-associated drug molecules. Macromolecular and small molecular model drugs (protein enzyme (horseradish peroxidase, HRP) and an antitumor drug (doxorubicin, DOX)) were also loaded in an SS/dextran hydrogel. These hydrogels resulted in sustained HRP release for over 50 days and a faster release of DOX with over 90% release at day 32 [76].

A simple way of modulating the drug delivery capacity is through SS-based constructs by adjusting its final concentration [77]. Higher concentrations promote drug entrapment efficiency as a result of an increase in the polar functional groups (hydroxyl, carboxyl) [77].

Long-term treatment and continuous drug delivery are usually suitable for chronic wounds, while more intermittent drug release profiles are useful in acute wounds or surgical incisions, where rapid healing without excessive drug exposure is required [78,79]. The bilayer system developed here allows a phased drug delivery approach with enhanced controlled release.

### 2.5. Sterility Tests

**Figure 6:**
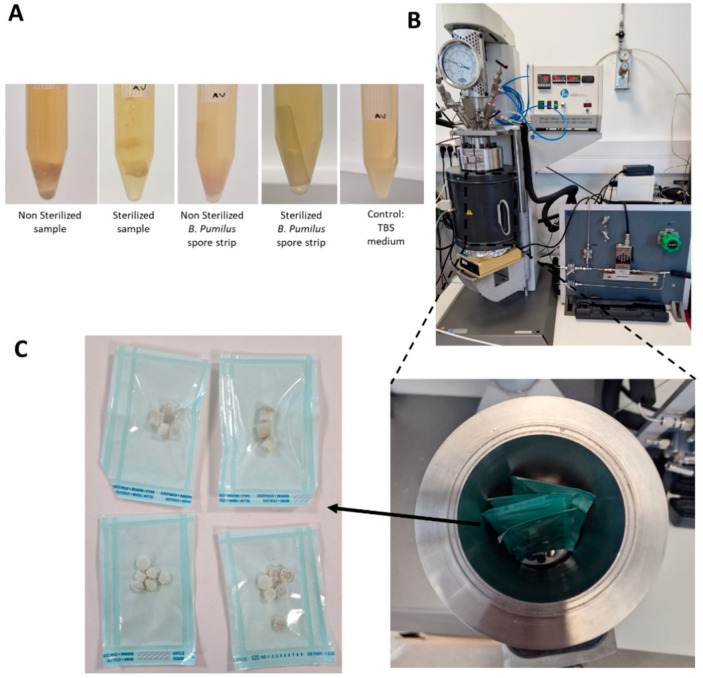
SCO_2_ sterilization of the silk-based wound dressings, (**A**) turbidity tests conducted after 14 days of incubation, (**B**) reactor used, (**C**) reactor vessel with samples stored in sterilization pouches, and (**C**) bilayers after sterilization.

The sterility tests and experimental sCO_2_ set-up are represented in Figure 6.

In addition to the physicochemical properties of the wound dressing, adequate sterilization is always a concern in the application of these biomaterials. Sterilization is one of the most challenging steps in biomedical engineering, especially in protein-based constructs, as it can lead to conformational changes in the proteins, compromising their performance in vitro [80]. This applies for example to protein-based hydrogels, which tend to denature when submitted to high temperatures [81]. Ethanol is reported to lead to changes in pore size, resulting from the shrinkage of SS scaffolds, and gamma ray irradiation can result in high degradation rates [80]. Some studies guarantee the sterilization of an SS gel by keeping the solution in a bioclean environment [82], which brings complexity to its production and transportation. Moreover, when using an SS solution, the shelf life is reduced when compared to a rehydrated construct. In our previous work, scCO_2_ sterilization of an SS powder was successfully achieved, without compromising the protein’s intrinsic gelling properties and its secondary structure (Madrid). Based on this, a new protocol was developed for the sterilization of SS and SF scaffolds. The turbidity tests performed using *B. pumilus* and samples immersed in tryptic soy broth (TSB) liquid medium at the optimal growth temperatures showed that after 14 days no contamination occurred for the sterilized samples as the medium remained clean (Figure 6A). The unprocessed materials resulted in turbidity after 24 h (Figure S1), indicating contamination of the samples. The capability of the sterilized scaffold to recover its hydrogel-like properties upon hydration without losing its stability opens room for the utilization of these materials in the dry state before use, with all the advantages related to preservation requirements, shelf life, or transportation logistics.

### 2.6. Biological Evaluation

**Figure 7:**
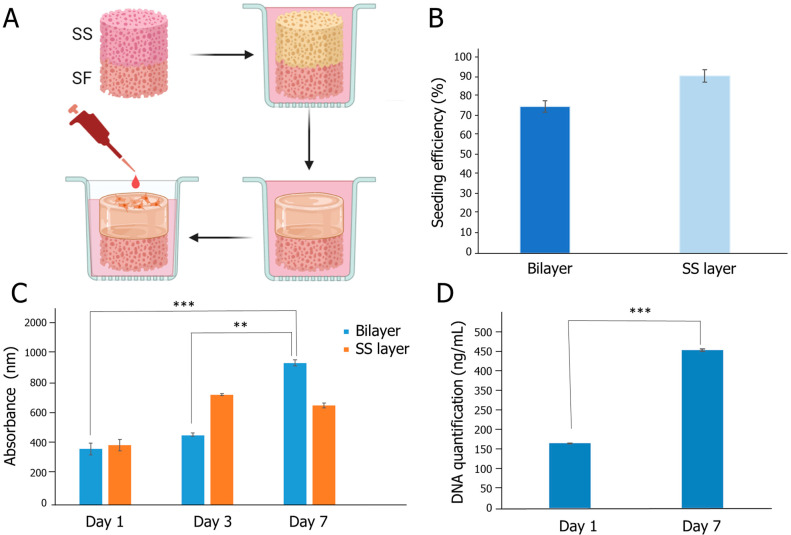
(**A**) Rehydration of the sterilized dry scaffold and reversion of SS layer to a hydrogel-like structure before cell seeding; (**B**) seeding efficiency on the bilayer and SS structures; (**C**) cell metabolic activity of HDFs seeded on the bilayer and SS structures; (**D**) DNA content on the bilayer constructs. Asterisks correspond to statistically significant differences (*** *p* < 0.001, ** *p* < 0.01).

The biological performance of the bilayer scaffold was evaluated after immersion in DMEM serum-free medium for 2 days, to guarantee that the SS layer adopted a hydrogel-like structure (Figure 7A).

The seeding efficiency on the constructs was higher than 75% (Figure 7B) and the metabolic activity of the HDFs increased over time on the bilayer structures (Figure 7C). Two-dimensional controls were used to verify the normal behavior of the HDFs (SI-2). Regarding the SS layer, which was used as a control, no significant differences were verified between 3 and 7 days of cell culture, suggesting that the SF layer contributed to the overall biological performance (Figure 7C). This is probably the result of the additional mechanical stability provided, which might be more conducive to cell attachment and growth. In addition, the varied pore size might offer better nutrient and oxygen permeability. The increase in cell proliferation was further corroborated through DNA quantification, which evidenced a significant increase in DNA content in the bilayer constructs after 7 days of culture (*p* < 0.001) (Figure 7D). The seeded structures were also imaged in confocal (SI-3), but due to the intrinsic fluorescence properties of silk [83], its visualization was hindered. The SEM images (SI-4) show that the cells are spreading in the SS matrix, supporting the in vitro tests carried out.

The interaction of the SS/SF bilayer scaffold with the wound environment was designed to optimize the healing process, highlighting its potential for future wound care applications. In the study, the SS layer, which is intended to be positioned directly against the wound, demonstrated significant swelling upon contact with exudate, achieving maximum swelling capacity within 1 h. This rapid swelling transforms SS into a hydrogel-like state, creating a moist and stable environment critical for cell growth and wound healing. The moist environment is sustained over time and results in enhanced in vitro cell proliferation. This is especially beneficial for wounds with low to medium exudate, as wounds with high exudate typically need synthetic sponges that have high absorption rates and are often managed with negative pressure therapy or additional secondary dressings [56,59].

Although studies on SS are still limited, previous studies from our group showed that the proposed SS–hydrogel formulation can promote cell adhesion, proliferation, and wound healing when applied in situ, i.e., directly into the wound of a db/db diabetic mouse model, promoting deposition of collagen fibers while having a local anti-inflammatory effect [33,55].

The external SF layer, designed to be the outer layer of the wound dressing, provides the necessary structural integrity and protection against external stresses due to load-bearing properties. The SF layer’s larger pores and crystalline structure make it robust enough to endure environmental factors while still allowing for oxygen exchange. The mechanical barrier in a skin defect also protects the wound from infection [84,85]. SF and silk-based wound dressings have been reported and are well documented [44]. According to Lee and co-authors [86], a woven silk textile applied as a wound dressing in rats with full-thickness burn wounds did not lead to inflammation up to 14 days post-injury and promoted wound healing when compared to non-treated animals. In addition, a randomized controlled clinical trial with 71 patients indicated that the SF film significantly reduced the time to wound healing and the incidence of adverse events compared to a commercial dressing [87]. Other works show that SF scaffolds promote epithelialization [88], keratinocyte migration, and fibroblast growth [89], tested on full-thickness mice models and ex vivo wound healing models undergoing elective surgery, respectively [10,11].

The SS/SF bilayer structure adopts a sponge-like behavior when rehydrated, which minimizes trauma upon removal. In addition, its sustained drug delivery can be interesting for long-term applications (from a few days to a week). The interconnected interface between the SS and SF layers ensures the overall integrity of the scaffold, maintaining high porosity and interconnectivity, a crucial feature of bilayer structures [90]. In a recent work by Kanokpanont et al. [91], both SS and SF were combined to develop a bilayer wound dressing. The wax non-adhesive layer-coated SF woven fabrics and SS–SF/gelatin were introduced as new wound dressings with the ability to reduce wound size and epithelialization in a full-thickness wound rat model. This configuration exhibited enhanced biological effectiveness in terms of cell growth and wound healing in an in vivo full-thickness wound model in rats while demonstrating reduced adherence to the wound in contrast to the commercially available 3M™ Tegaderm™ (3 M Corporate Headquarters, Maplewood, MN, USA), a known dressing composed of a thin polyurethane membrane coated with a layer of an acrylic adhesive. Furthermore, the SS/SF/gelatin layers attached to the wax-coated SF bilayer porous scaffold dressing promoted the synthesis of collagen type III, and a substantial presence of macrophages was observed on these dual-layered wound dressings [91]. Further clinical investigation carried out by Hasatsri et al. [19] proved that dressings were potentially useful in treating split-thickness skin graft donor sites. In this work, patients with split-thickness skin graft donor sites were evaluated in terms of systemic reactions, skin barrier functions, healing time, and pain scores, compared to Bactigras™ (Smith & Nephew, Watford, UK)(composed of leno weave gauze, white soft paraffin, and chlorhexidine acetate). The bilayer wound dressings exhibited accelerated healing compared to Bactigras, which was attributed to the combined properties of SF, SS, and gelatin. Moreover, the wound sites treated with the bilayer wound dressing showed significantly less pain and more rapid skin functional barrier recovery than those treated with Bactigras.

Other works focus on the development of bilayer wound dressings to achieve better regeneration of both the dermal and epidermal layers [92]. Boucard and colleagues [92] introduced a chitosan-based bilayer scaffold, composed of a soft and flexible lower hydrogel that was able to adapt and adhere to the wound site, and a rigid and dense layer to guarantee protection and gas exchange. When used in vivo in a pig animal model, the scaffold promoted collagen I and IV deposition as well as angiogenesis and migration of inflammatory cells into the wound site which is critical for the healing process, particularly in the proliferation and remodeling phases, leading to the formation of new tissue [92].

The potential of the SS/SF bilayer scaffolds for clinical use was assessed by scaling up the process to obtain a scaffold size of 30 mm diameter (Figure S5). Wound dressings are used to treat injuries of varying sizes and shapes. Thus, to have scalable processes that are aligned with the practical requirements of the medical device industry is important. The scale-up can also offer significant improvement in drug loading for wound healing applications. With larger batch sizes, enhanced drug loading efficiency and consistency across both layers of the scaffold can be achieved, ensuring consistent therapeutic effects [93]. Drug entrapment can be achieved while the layers are still in the gel state, which enables a more uniform distribution throughout the scaffold. Although the lyophilization process is reported to influence the release kinetics of the drug, it can also protect sensitive drugs from the degradation that might occur in harsher processing conditions [94].

This study presents a novel approach in the field of wound care through the development of an SS/SF bilayer scaffold. The integration of SS and SF in a bilayer configuration represents a significant advancement due to their distinct yet complementary properties. Notably, traditionally discarded SS, was processed into a hydrogel layer that promotes a moist wound environment and supports controlled drug delivery, as evidenced by our findings. This innovative use of SS alongside SF, which is known for its structural integrity and prolonged drug release capabilities, offers a dual-phase system tailored to meet the dynamic needs of wound healing. Moreover, our study introduces a scalable fabrication process using supercritical technology for sterilization, enhancing the material’s feasibility for clinical translation. The demonstrated biocompatibility, mechanical stability, and enhanced biological behavior of the SS/SF bilayer scaffold underscore its potential as a versatile and effective solution in biomedical therapeutics and tissue engineering. As we look ahead, the natural progression of this work involves a comprehensive exploration of the bilayer dressing performance in vitro, to understand the mechanisms behind the cell behavior and in vivo and to evaluate the wound healing efficacy. Additionally, its drug-delivery capacity should be explored in the future. Parameters such as wound closure rates, tissue regeneration, and inflammatory response should be assessed, allowing a direct comparison with existing wound dressings. To gain a deeper understanding of the dressing’s long-term biocompatibility, histological analyses of healed tissue are relevant. This involves scrutinizing the integration of the dressing with host tissue, monitoring the inflammatory response, and assessing overall tissue morphology over extended periods. Beyond its in vitro mechanical evaluation, the dressing’s performance in in vitro dynamic conditions should be explored to ensure its mechanical stability and durability under circumstances that mimic real-life scenarios, particularly the kinetics of low/medium exudative wounds.

## 3. Conclusions

In the past decade, there have been significant advancements in both the creation of natural-based materials and their utilization in modern wound dressings. Nevertheless, existing dressings still face challenges, including complex manufacturing procedures and a lack of scientific validation.

In this study, we propose a simple method for developing natural-based bilayer wound dressings. This approach leverages the intrinsic properties of both SS and SF proteins, without the need for additional materials. This not only simplifies the manufacturing process but also aligns with eco-friendly practices. The SS/SF bilayer scaffolds embody a new approach to wound care, with the potential to be scalable and used in full-thickness wounds as a dried, off-the-shelf wound dressing, sterilized through scCO_2_. After lyophilization, the resultant structure exhibited a high degree of porosity (>80%), displaying uniform and strongly interconnected layers. The SS layer, which would be positioned directly against the wound, features smaller pores (≈20–50 µm) and a hydrogel-like composition after re-hydration that maintains a moist healing environment, which is critical for optimal wound healing. Its high swelling capacity can efficiently absorb wound exudate while acting as a fast drug delivery system directly to the wound site. On the other hand, the SF scaffold layer, the wound dressing’s outermost layer, with larger pores (≈100 µm) and a crystalline structure, offers structural integrity, support, and protection against external stresses. SF’s slow drug release profile opens new possibilities for a dual-phase drug delivery approach. All combined, these silk-based structures enhanced cell activity for wound healing, as demonstrated by the HDFs cultured up to 7 days. According to the characteristics of the wound, the presented biomaterials can be tailored to fit different requirements, by adjusting the SS/SF ratio and scaling up the process.

Overall, the developed SS/SF bilayer scaffolds have the potential to be used as a sustainable wound dressing material for deep and exudative wounds and as a drug carrier with dual-release capacity.

## 4. Materials and Methods

### 4.1. Scaffold Production

#### 4.1.1. Sericin Extraction and Processing

SS processing was achieved based on a patented methodology, which enables the preservation of the SS conformation structure and the resulting physicochemical and biological properties after degumming, concentration, and sterilization. Briefly, a total of 10 g of Bombyx mori cocoons (APPACDM Sericulture, Castelo Branco, Portugal) were cut and cleaned before immersion in 1 L of deionized water brought to a boil. The fibers were allowed to remain in the boiling water for approximately 1 h and 30 min, resulting in a remaining volume of 200 mL of SS solution. The solution was subsequently filtered and promptly frozen using liquid nitrogen, followed by storage at −80 °C until lyophilization (Off-The-Shelf Silk Sericin; Methods and Uses Thereof, Patent nr 2023231000146).

#### 4.1.2. Fibroin Extraction and Processing

To obtain purified SF, silk cocoons were immersed in a boiling solution of sodium carbonate (0.02 M) for a duration of 1 h. Subsequently, the fibers were rinsed thoroughly with distilled water to completely remove the degumming solution. Next, the purified SF was dissolved in a lithium bromide solution (9.3 M) for 1 h at a temperature of 70 °C. The resulting solution was then dialyzed against distilled water for 48 h using benzoylated dialysis tubing with a molecular weight cutoff (MWCO) of 2 kDa. To concentrate the SF, the solution was subjected to concentration against a 20 wt% poly(ethylene glycol) solution for a minimum of 3 h. The concentration of the SF solution was determined by measuring the dry weight of the SF solution after being placed in an oven at 70 °C overnight. The prepared SF solution was stored at 4 °C until further use.

To crosslink SF, a 7 wt% solution was combined with enzyme horseradish peroxidase (HRP) (type VI, 0.84 mg mL^−1^, Sigma-Aldrich, St. Louis, MO, USA) and a hydrogen peroxide solution (H_2_O_2_, 0.36 wt%; Panreac, Barcelona, Spain) based on previous works [95,96]. This was followed by a salt-leaching process to obtain the porous SF-based scaffolds, in which a 1 mL SF/HRP/hydrogen peroxide(H_2_O_2_) solution was transferred into a cylindrical-shaped silicone mold (9 mm inner diameter). To generate β-sheet-induced SF structures, the following steps were undertaken: 2 g of granular sodium chloride with a particle size of 200 µm were slowly added to the SF solution. The sodium chloride particles were gently introduced into the silicon molds to facilitate salt precipitation. Subsequently, the molds containing the mixture were placed in an oven set to 37 °C, allowing the enzymatic reaction to proceed. Over 72 h, the undissolved sodium chloride particles acted as a porogen and were gradually leached out by soaking the molds in distilled water. This resulted in the formation of HRP-crosslinked SF scaffolds. Finally, the scaffolds were extracted from the molds using a biopsy punch with an 8 mm inner diameter (Kai biopsy punch) [39,95].

#### 4.1.3. SS/SF Bilayer Assembly

**Figure 8:**
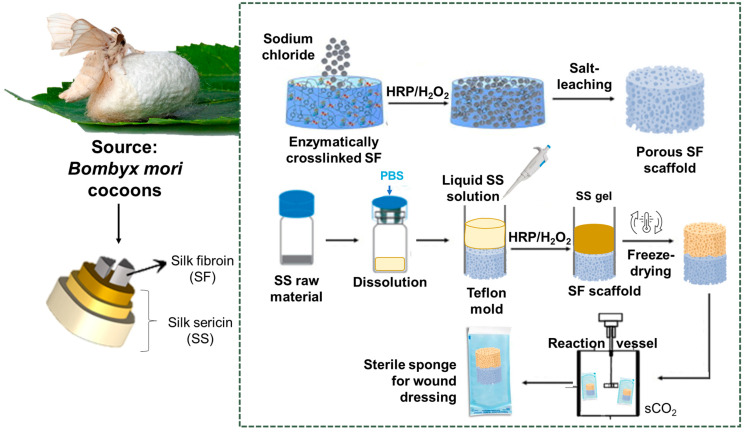
Illustration of silk cocoon composition, SS/SF bilayer assembly, and sCO_2_ sterilization to obtain a ready-to-use wound dressing. Reprinted (adapted) with permission from [39]. Copyright {2019} American Chemical Society.

The obtained SS powder was dissolved in boiling phosphate-buffered saline solution (PBS, 2.5 wt%), combined with HRP (2 mg/mL, 5 µL/mL SS)/H_2_O_2_ (30%, 50 µL/mL SS), as previously described [55], and poured to the top of the SF scaffold placed in the mold (Figure 8). After 2–3 h incubation, the hydrogel/scaffold structure was frozen in liquid nitrogen and lyophilized to obtain a dry and silk bilayer scaffold. Samples with dimensions of 0.6 cm in width and 1 cm in length, maintaining a 1:1 ratio of SF to SS, were analyzed.

#### 4.1.4. Supercritical CO_2_ (sCO_2_) Sterilization

Sterilization was achieved using supercritical fluid CO_2_. This technology has previously been demonstrated to be effective in sterilizing SS and SF membranes without altering their physicochemical properties [97]. Sterilization pouches (Tyvek, Richmond, VA, USA) with the samples were placed inside a pressure vessel of a 1.2 L stainless steel autoclave (Parr Instrument Company, Moline, IL, USA). Premium CO_2_ Liquid Premier with 99.995% purity was introduced into the pressure vessel via a high-pressure pump at 50 g/min, and the pressure was set to 140 bar. The temperature was adjusted to 40 °C and the rotation motor speed was set at 600 rpm. H_2_O_2_ (30%, Sigma Aldrich, St. Louis, MO, USA) was used as a co-additive at a concentration of 300 ppm. After 4 h of batch operation, the vessel was dried in semi-continuous mode for 1 h before depressurizing for 30 min using a manually operated valve. After treatment, the samples were stored in the desiccator.

### 4.2. Physicochemical Characterization

#### 4.2.1. Fourier Transform Infrared Spectroscopy (FTIR) Analysis

The analysis of the functional groups in the various SS and SF layer solutions was carried out using Fourier Transform Infrared Spectroscopy (FT/IR-6200 Spectrometer (Jasco, Easton, MD, USA)) with a PerkinElmer Spectrum instrument. After assembling the SS/SF bilayer structure, and lyophilizing the resulting 3D construct, the dry material was analyzed.

To obtain the spectra, the samples were dried, and measurements were taken in the range of 4000−400 cm^−1^ with a resolution of 2 cm^−1^ using 4 accumulations. Before each measurement, a background spectrum was collected under the same operational conditions. To ensure accuracy, baseline-point adjustment, and spectra normalization were conducted on the acquired data.

#### 4.2.2. Scanning Electron Microscopy (SEM), Cryo-Scanning Electron Microscopy (Cryo-SEM), and Energy Dispersive Spectroscopy (EDS)

To perform SEM in the dry bilayers, the materials were dehydrated before analysis using increasing concentrations of ethanol solutions (10%, 30%, 50%, 70%, 90%, 100% *v*/*v*) for 15 min [98]. Cryo-SEM was conducted in the lyophilized samples after immersion in ultrapure water for 3 h and 3 days to allow the transition of the SS scaffold to a hydrogel-like structure and to evaluate the structure over time using a high-resolution scanning electron microscope equipped with X-ray microanalysis and CryoSEM capabilities: JEOL JSM 6301F/Oxford INCA Energy 350/Gatan Alto 2500. The specimens were rapidly cooled by immersing them in sub-cooled nitrogen (slush nitrogen) and subsequently transferred under vacuum to the cold stage of the preparation chamber. Following this, duplicate samples of different SS specimens were fractured, sublimated (etched) for 300 s at a temperature of −90 °C, and coated with Au/Pd using sputtering for a duration of 48 s. Subsequently, the samples were transferred to the SEM chamber and examined at a temperature of −150 °C.

SEM/EDS analysis was also conducted in the re-hydrated samples for elemental analysis and to assure sample purity.

#### 4.2.3. Microcomputed Tomography (Micro-CT)

The SS, SF, and SF/SS bilayers were scanned in micro-CT equipment (SkyScan 1174; Bruker, Brussel, Belgium) with the following parameters: 50 kV of voltage, a current of 800 mA, an image pixel size of 11.67 µm, exposure time of 6000 ms, and a rotation step of 0.8°. The three-dimensional reconstructions were made using CTan (version 1.20.3.0) and CTvox (version 3.2.0) software (Bruker, microCT, Kontich, Belgium), while the transversal plane views were made using Dataviewer. Porosity measurements (*n* = 3) were derived from micro-computed tomography (µ-CT) reconstructions.

#### 4.2.4. Mechanical Properties

Compression tests were conducted using a Discovery HR20 Rheometer (TA Instruments) equipped with a stainless steel parallel plate setup (diameter: 20 mm, gap: 1 mm), and data acquisition was performed using the TRIOS software (version 5.0). The specimens’ dimensions were 4 mm in height and 4 mm in diameter. The compressive properties were determined using a 7 mm displacement range and a displacement rate of 5 mm·min^−1^. The Young’s modulus was measured from the tangent slope of the stress–strain curve (within the elastic regime). Three samples were tested in both dry and hydrated conditions, from which the average value was taken.

$$E=\frac{\Delta \sigma }{\Delta \varepsilon },\;\mathrm{where}\;\sigma \;\mathrm{is}\;\mathrm{the}\;\mathrm{stress}\;\mathrm{and}\;\varepsilon \;\mathrm{the}\;\mathrm{strain}\text{.}$$

#### 4.2.5. Drug Delivery Capacity

In this study, the release profile of the albumin-fluorescein isothiocyanate conjugate (albumin-FITC) (model drug) from the produced bilayer scaffolds and the SS and SF scaffold was investigated. First, the scaffolds were hydrated in PBS for 12 h after preparation. Subsequently, the scaffolds were immersed overnight at room temperature (1.5 mL/scaffold) in a 100 μg/mL solution of albumin-FITC. Following immersion, the scaffolds were rinsed in PBS. The albumin-FITC release profiles of the as-prepared scaffolds were evaluated by immersion of each specimen in 4 mL PBS. The release of albumin-FITC was tested at 2, 4, 6, 24, 48, 72, 120, and 168 h. At each time point, the supernatant from each specimen was removed and an equal volume of fresh PBS was added. For the quantification of the released albumin-FITC, the fluorescence intensity of 100 μL of the supernatant of the removed PBS was read by a microplate reader (Synergy HT, Bio-Tek, VT, USA), with the excitation wavelength at 485/20 nm and the emission wavelength at 528/20 nm. Scaffolds without albumin-FITC incorporation were used as controls. Five specimens were used for each group.

#### 4.2.6. Swelling Properties

The bilayers (4 × 4 mm) were then immersed into 2 mL of phosphate-buffered saline (PBS) (pH = 7.4) at 37 °C. The weights of all the samples were recorded before (Wd) and after immersion at regular intervals (Ww) (1, 3, 6, 12, 48, 69, 93, 117, and 168 h). Subsequently, the samples were dried, and the dry weights were measured (Wd). The swelling ratio was calculated as (%) = (Ws − Wd)/Wd × 100.

#### 4.2.7. Sterility Assessment

Turbidity tests were performed to assess the sterility of the materials and of the process. Commercial spore strips with 106 spores of the biological indicators of *Bacillus pumilus* (*B. pumilus* ATCC 27142 Valencia, Spain) were purchased from Sigma-Aldrich. The treated materials and spore strips were incubated in 6 mL of TSB liquid medium at the optimal growth temperatures (37 °C), and the bacterial growth was visually evaluated after 1, 3, 7, and 14 days.

### 4.3. In Vitro Biological Assessment

#### 4.3.1. Cell Culture

Human dermal fibroblasts (HDFs) were sourced from Lonza (catalog #CC-2509) and cultivated as a monolayer under standard conditions of 37 °C and 5% CO2. The basal medium used was Dulbecco’s Modified Eagle’s Medium (DMEM; Gibco™ DMEM, high glucose), supplemented with 10% fetal bovine serum (FBS; BioWest, Nuaillé, France) and 1% Penicillin-Streptomycin solution (Lonza, Basel, Switzerland). Once the cells reached confluence, they were detached from the culture flasks using 0.25% trypsin (Gibco, Thermo Fisher Scientific, Waltham, MA, USA), followed by centrifugation and resuspension in culture medium.

#### 4.3.2. Micro Seeding

The sterilized bilayer and SS scaffolds were immersed in FBS-free DMEM for 48 h prior to cell seeding. Before adding the HDFs, the medium was collected, and the bilayers moved to a new 12-well plate; 15 µL of concentrated cell suspension was added to the scaffolds (1 × 10^5^ cells/scaffold), followed by 100 µL of DMEM. After 1 h of incubation, the remaining medium (2 mL) was added to the scaffolds and maintained in culture for up to 7 days.

#### 4.3.3. Cell Metabolic Activity

Metabolic activity in silk bilayers was evaluated using the AlamarBlue^®^ reagent (AB). At designated time points (1, 3, and 7 days), each well received 2 mL of a 10% (*v*/*v*) AB solution in phenol red-free medium. Following a 5 h incubation period, 100 µL of the AB solution was transferred in triplicate to a 96-well cell culture plate (Greiner Bio-one, Frickenhausen, Germany). Absorbance measurements were taken using Biotek Synergy HT plates (BioTek Instruments, Winooski, VT, USA) at an excitation wavelength of 560 nm and an emission wavelength of 590/35 nm. The data were analyzed and presented as mean ± standard deviation, with a sample size (n) of 8 for each data point. Statistical analysis was conducted using GraphPad Prism 8.0 (GraphPad Software). The two-way ANOVA test followed by Tukey’s method was employed as a multiple comparison post-test. Significance levels were denoted as * *p* < 0.05, ** *p* < 0.01, *** *p* < 0.001, **** *p* < 0.0001.

#### 4.3.4. Fluorescence Staining

The seeded structures were fixed by immersing them in a histological tissue fixative solution (10% Formalin, Sigma Aldrich, St. Louis, MO, USA) for 2 h. Following fixation, the samples were washed with phosphate-buffered saline (PBS) and then stored in a refrigerated environment until staining. For immunostaining, permeabilization buffer (1% *v*/*v* TritonTM X-100, Sigma-Aldrich, St. Louis, MO, USA) was applied to cover the samples for 10 min. Afterwards, the samples were rinsed three times with PBST (1% *v*/*v* TWEEN^®^ 20, Sigma-Aldrich, St. Louis, MO, USA). To prevent non-specific binding, a blocking buffer solution (1:10, Abcam) was then added for 30 min, followed by the addition of specific fluorescent markers. Phalloidin (Alexa Fluor 594 phalloidin) (Invitrogen, Carlsbad, CA, USA) was added at a dilution of 1:400 for 45 min, and DAPI (Thermo Fisher scientific, Waltham, MA, USA) was added at a dilution of 1:1000 for 10 min. Between the addition of each dye, the samples underwent three washes with PBST for 5 min each and were stored in the refrigerator until analysis. Bright-field and fluorescent images were acquired using a Keyence BZ-X700 microscope along with the associated BZ-X Analyzer software.

#### 4.3.5. Cell Proliferation

To perform DNA quantification (Invitrogen Quant-iTTM PicoGreenTM dsDNA Assay Kit, Carlsbad, CA, USA), the samples were collected at two time points: after 1 day and 7 days of cell seeding. The samples were collected in water and frozen using liquid nitrogen to halt any biological processes and maintain the sample integrity until further analysis. On the day of analysis, the frozen samples were thawed by placing them in a thermostatic bath at 37 °C for 1 h. Following the thermostatic bath treatment, the samples were subjected to sonication using a sonicator probe at 30% intensity for 2 min. Sonication was employed to disrupt the scaffold structure, promote cell lysis, and release the nucleic acids for quantification using the Picogreen assay. The Picogreen assay is a sensitive and reliable method for quantifying dsDNA (double-stranded DNA) in a sample. The assay is based on the selective binding of Picogreen dye to dsDNA, resulting in a fluorescence signal that is proportional to the amount of dsDNA present. To perform the Picogreen assay, a solution was prepared from the sonicated sample, which contained the released nucleic acids. Briefly, an aliquot of the sonicated sample was taken and mixed with an appropriate volume of the Picogreen working solution, according to the manufacturer’s instructions. The mixture was then incubated in the dark at room temperature for a specified period, as recommended by the manufacturer. After the incubation period, the fluorescence of the Picogreen–dsDNA complex was measured using a fluorescence spectrophotometer or a plate reader with appropriate excitation and emission wavelengths for the Picogreen dye. The fluorescence intensity was recorded, and the DNA concentration in the sample was calculated using a standard curve generated with known concentrations of dsDNA standards. The obtained DNA concentrations were then analyzed to assess the changes in DNA content at the 1-day and 7-day time points, providing insights into the cellular proliferation within the bilayer scaffold. The data were analyzed and presented as mean ± standard deviation, with a sample size (n) of 8 for each data point. Statistical analysis was conducted using GraphPad Prism 8.0 (GraphPad Software), as previously described.

## Data Availability

The data presented in this study are available on request from the corresponding author (ensuring confidentiality and protecting intellectual property).

## Supplementary Materials

The following supporting information can be downloaded at: https://www.mdpi.com/article/10.3390/gels10070439/s1, Figure S1: Turbidity tests conducted after 24 h of incubation for the non-sterilized material, Figure S2. Cell metabolic activity of HDFs seeded on the bilayer, SS structures and 2D controls. Figure S3. Confocal images of the bi-layer structure (red: phalloidin, blue: DAPI). Figure S4. SEM images: (A) Control Sericin, (B) Bilayer after 1 (B1) and 7 days (B2) of cell culture. Figure S5. Scale up process for the proposed biomaterial.
