# Supplementary material for: Streamlining Skin Regeneration: A Ready-To-Use Silk Bilayer Wound Dressing
Source: Gels. 2024 Jun 30;10(7):439. doi: 10.3390/gels10070439 (PMC11276312; doi:10.3390/gels10070439)
Supplement: Supplementary file 1 [file gels-10-00439-s001.zip › gels-3027846-supplementary.pdf]

Supplementary Material (S)

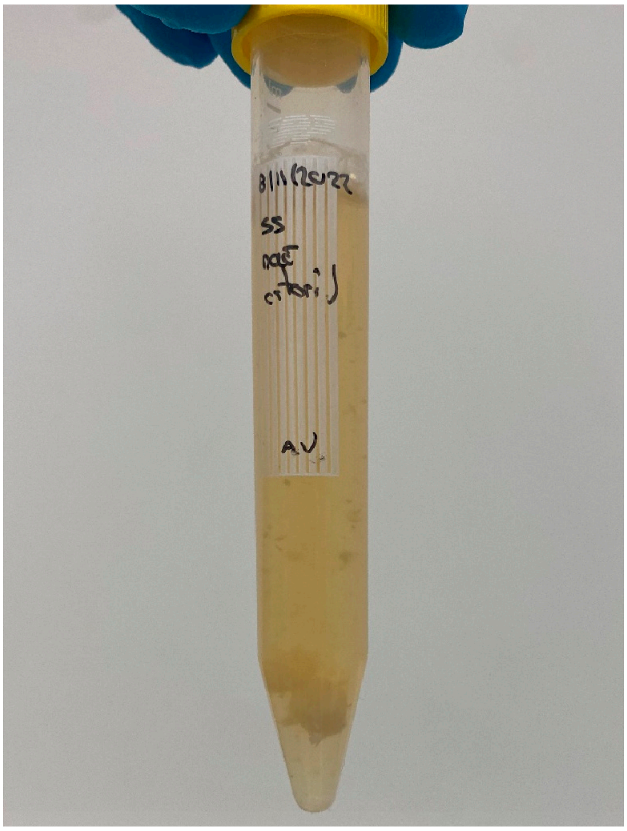

Figure S1. Turbidity tests conducted after 24h of incubation for the non-sterilized material.

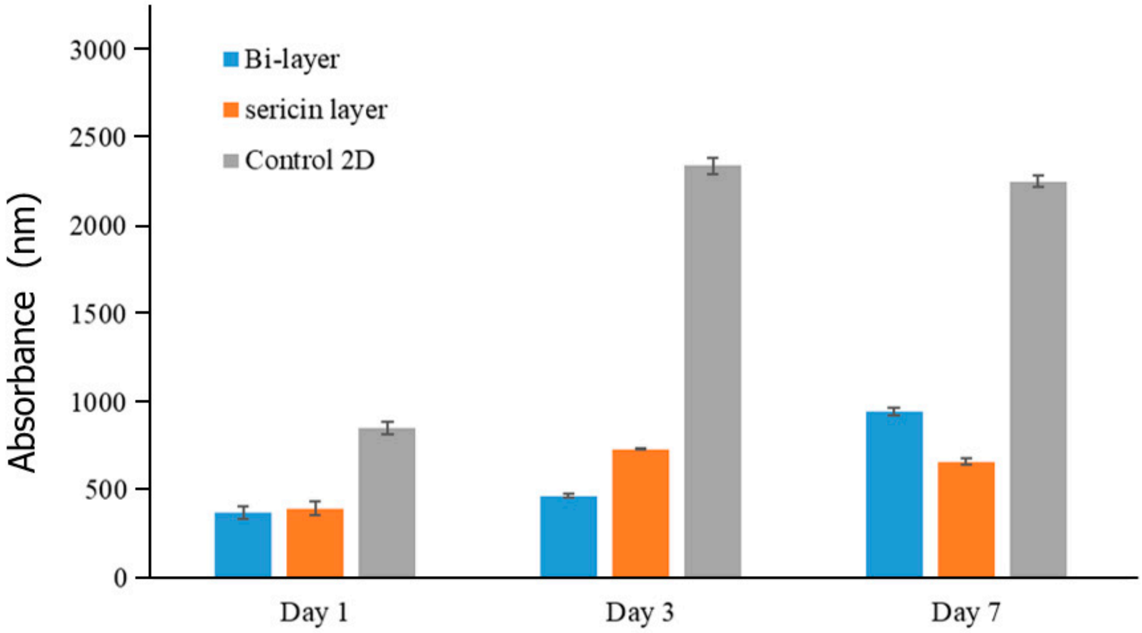

Figure S2. Cell metabolic activity of HDFs seeded on the bilayer, SS structures and 2D controls.

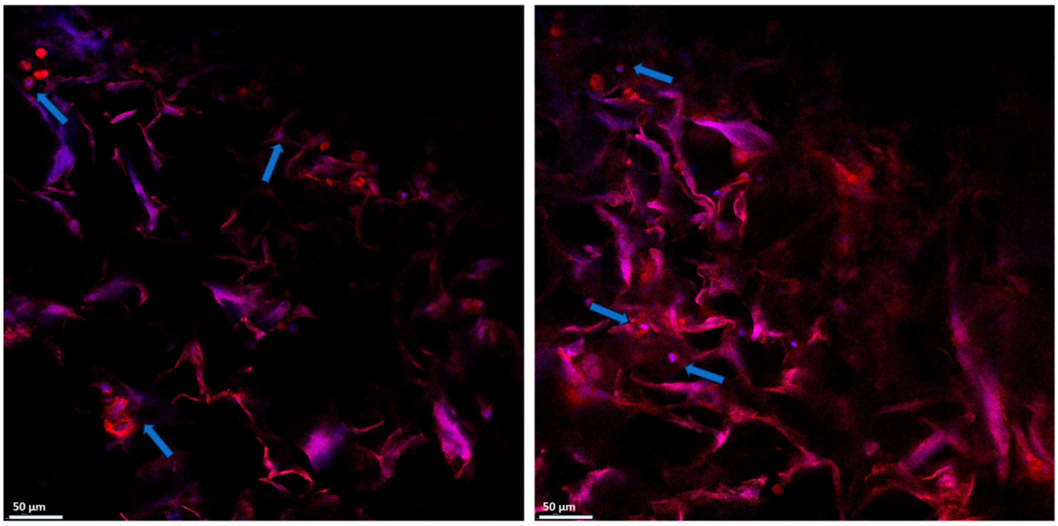

Figure S3. Confocal images of the bi-layer structure (red: phalloidin, blue: DAPI).

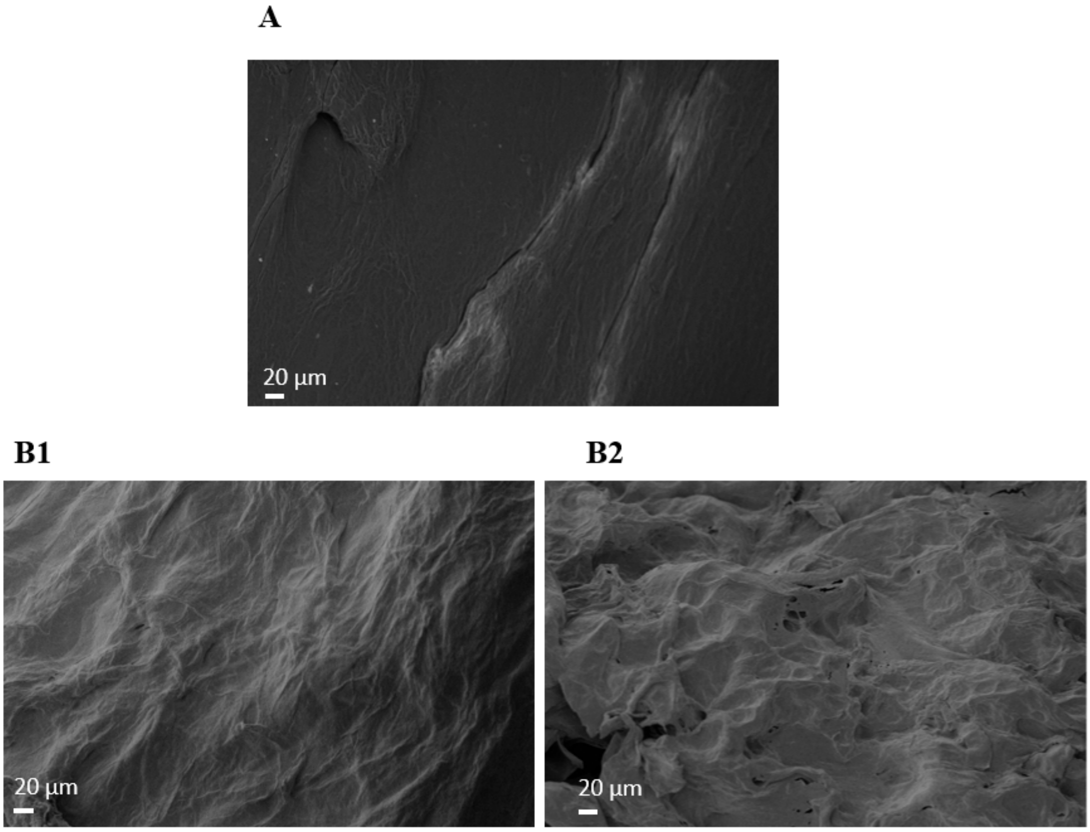

Figure S4. SEM images: A) Control Sericin, B) Bilayer after 1 (B1) and 7 days (B2) of cell culture.

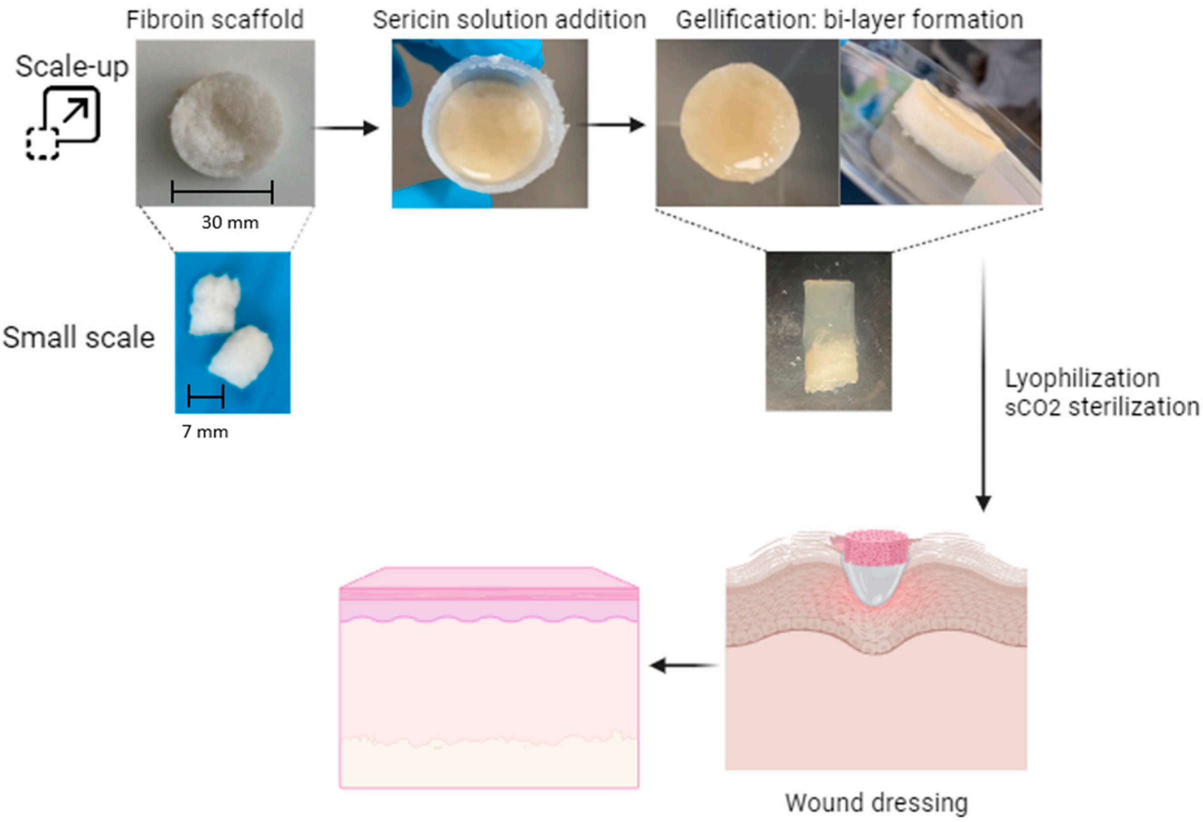

Figure S5. Scale up process for the proposed biomaterial.
